# Analysis and Testing of a Flyable Micro Flapping-Wing Rotor with a Highly Efficient Elastic Mechanism

**DOI:** 10.3390/biomimetics9120737

**Published:** 2024-12-03

**Authors:** Yingjun Pan, Huijuan Su, Shijun Guo, Si Chen, Xun Huang

**Affiliations:** 1 Centre for Aeronautics, Faculty of Engineering and Applied Sciences, Cranfield University, Bedford MK43 0AL, UK; s.guo@cranfield.ac.uk (S.G.); xun.huang@cranfield.ac.uk (X.H.); 2 Faculty of Arts, Science and Technology, University of Northampton, Northampton NN1 5PH, UK; huijuan.su@northampton.ac.uk; 3 College of Mechanical and Electrical Engineering, Wenzhou University, Wenzhou 325035, China; 20200316@wzu.edu.cn

**Keywords:** flapping-wing microrotor, elastic mechanism, aerodynamics, dynamic analysis, power efficiency

## Abstract

A Flapping-Wing Rotor (FWR) is a novel bio-inspired micro aerial vehicle configuration, featuring unique wing motions which combine active flapping and passive rotation for high lift production. Power efficiency in flight has recently emerged as a critical factor in FWR development. The current study investigates an elastic flapping mechanism to improve FWRs’ power efficiency by incorporating springs into the system. The elastic force counteracts the system inertia to accelerate or decelerate the wing motion, reducing the power demand and increasing efficiency. A dynamic model was developed to simulate the unique kinematics of the FWR’s wing motions and its elastic mechanism, considering the coupling of aerodynamic and inertial forces generated by the wings, along with the elastic and driven forces from the mechanism. The effects of the spring stiffness on the aerodynamic performance and power efficiency were investigated. The model was then verified through experimental testing. When a spring stiffness close to the mechanical system resonance was applied, the power efficiency of the test model increased by 16% compared to the baseline model without springs, generating an equivalent average lift. With an optimal elastic flapping mechanism for greater lift and lower power consumption, the FWR was fully constructed with onboard power and a control receiver weighing 27.79 g, successfully achieving vertical take-off flight. The current model produces ten times greater lift and has nearly double the wing area of the first 2.6 g flyable FWR prototype.

## 1. Introduction

Flapping-wing micro aerial vehicles (MAVs), which mimic insect flight and are characterised by remarkable manoeuvrability, agility, and hovering capability, exemplify the sophisticated mechanisation of miniature-scale flying systems using unsteady aerodynamics [1,2]. These systems inherently face challenges in their design, manufacture, and efficiency, particularly due to limited onboard energy storage for adequate flight endurance. Their reduced aerodynamic efficiency at low Reynolds numbers and limited energy capacity hinder flapping-wing MAVs from achieving long loiter or hovering times of over 60 min [3,4,5,6,7,8,9,10,11]. For example, the 28.2 g Delfly Nimble developed by Karásek et al. [11], while representing significant progress, has a hovering time of 5 min. Efforts to increase the power supply have not substantially improved the flight endurance due to the considerable energetic demands of their mechanisms [12]. Similarly, Zhan et al. [5] developed a 20.4 g Hummingbird robot that employed a dual-motor system and achieved a flight time of 20 s before losing control. In comparison, the 19 g Nano Hummingbird, with a single motor and similar wingspan and weight, achieved a maximum flight endurance of 4 min [8]. Besides the high energy cost of the flapping mechanism, the intrinsic low aerodynamic efficiency of flapping wings also contributes to these limitations.

To enhance the lift and power efficiency, researchers have made efforts to develop novel Flapping-Wing Rotor (FWR) MAVs in recent years [13,14,15,16]. FWRs, incorporating active flapping and passive rotation, exhibit considerable improvements in aerodynamic performance compared to conventional insect-like flapping wings [13]. FWRs generated more than double the lift of rotary wings at equivalent power efficiency levels in a theoretical study [14]. A 30 g three-winged FWR achieved free flight, marking significant progress despite challenges with sustained flight due to controllability and power constraints [15]. Another 22.7 g two-winged FWR with passive pitching achieved vertical take-off and free flight [16]. Passive wing pitching effectively achieved nearly optimal kinematics of the flapping motion and enhanced aerodynamic efficiency [17,18], making the two-winged model more aerodynamically efficient than its three-winged counterpart [15]. However, this model required a 9V input from a ground-based DC source for flight tests, rather than utilising an onboard battery. With recent advances in onboard-powered FWR flight, power efficiency and effective power utilisation are becoming critical factors.

Insects utilise elastic storage, such as their flight muscles, thorax, and wing hinges, to minimise their flight energy consumption [19,20]. The inertial cost of wing acceleration is eliminated through energy storage during wing deceleration in the previous stroke, resulting in the total mechanical power required for insect flight being solely for aerodynamic forces [21]. Conventional flapping-wing MAVs have identified two strategies for mimicking such elastic storage, either by implementing an entirely elastic flapping mechanism [22] or by introducing an elastic component in a motor-driven rigid body mechanism [23,24,25,26]. In the latter case, Beak et al. [27] demonstrated an efficient resonant drive for a conventional flapping-wing robot with a motor-driven slider-crank mechanism, achieving a 19% power reduction at 16 Hz compared to a spring-less system at the same frequency in air. However, the application of springs in FWRs remains relatively unexplored. FWRs featuring asymmetrically installed wings undergo passive rotation around the central body axis due to the active flapping motion, which enhances the lift and reduces the required power input. These unique kinematics of motions advance the conventional flapping-wing MAVs. The current study investigates the impact of an elastic mechanism on the coupled motions of FWRs and evaluates the potential power savings. Such savings are essential for advancing FWR-MAV design. By focusing on the unique aspects of FWRs and the underexplored area of spring implementation to enhance FWRs’ flight capabilities, this study aims to provide meaningful contributions to the field.

This paper develops a dynamic model of the FWR mechanism combined with a quasi-steady aerodynamic wing model (Section 2.1) to analyse the nonlinear behaviours of the system (Section 2.2). This includes examining the effects of the spring stiffness on the kinematics of the FWR’s motion and aerodynamic and inertial forces. In Section 3.1, a physical model of the FWR-MAV is constructed for force measurements. By comparing the force production in the FWR model with varying springs against the input power at different motor speeds (flapping frequencies), the relationship between the spring stiffness and power efficiency is established (Section 3.2 and Section 3.3). More importantly, the FWR is reconstructed to incorporate an onboard power and control device for take-off flight tests (Section 4). Our principal findings are summarised in the Conclusions (Section 5).

## 2. The Spring-Integrated Flapping-Wing Rotor and Analysis Method

**Figure 1:**
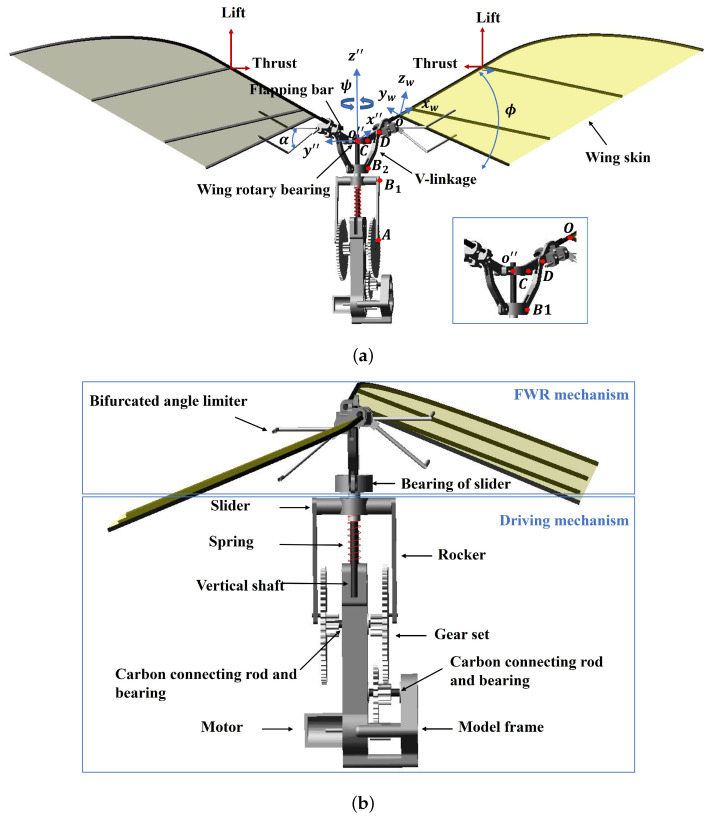
The FWR model of flapping and rotating motions about a shaft with a spring: (**a**) front view and (**b**) side view.

The FWR shown in Figure 1 is driven by a 3.7V DC motor operating at 4000 rpm and incorporates a 19.5:1 double-reduction gear unit. The parameters of the components are presented in Table 1. A pair of wings with a span-wise length of 330 mm are mounted on a vertical shaft via a V-shaped linkage in an axially symmetric orientation around the centre. Each wing span beam is inserted into a sleeve unit at the end of a flapping bar that is connected to the shaft and the V-shaped linkage. The bifurcated angle limiter unit enables the wing to execute passive pitching motions within a predetermined range, subject to the aerodynamic and inertial forces. The wing generates lift and thrust during the flapping motion due to the inverse Kármán vortex. The opposing thrusts of the two wings produce rotational motions around the central shaft. The three rigid-body rotation motions of the FWR are denoted by rotating, flapping, and pitching angles (ψ, ϕ, and α, respectively).

### 2.1. Aerodynamic Model of an FWR Wing

**Figure 2:**
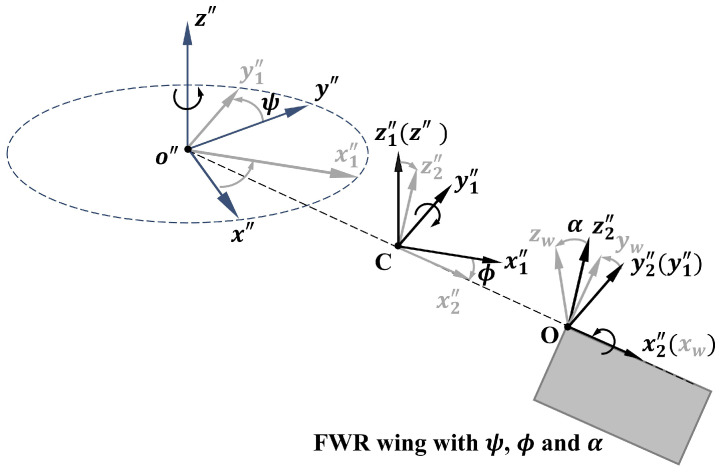
Coordinate systems and wing motions of the FWR wing.

Figure 2 presents the definitions of the coordinate systems used for the FWR wing. The wing rotation plane stays horizontal during hovering flight. The rigid-body rotations incorporate the following frames: The inertial frame $x^{\prime \prime }y^{\prime \prime }z^{\prime \prime }$ is positioned at point $o^{\prime \prime }$ on the vertical shaft. The co-rotating frame $x_{1}^{\prime \prime }y_{1}^{\prime \prime }z_{1}^{\prime \prime }$ is aligned with the $z^{\prime \prime }$-direction of the inertial frame at point *C*. The intermediate frame $x_{2}^{\prime \prime }y_{2}^{\prime \prime }z_{2}^{\prime \prime }$ represents the flapping motion. The wing-fixed frame $x_{w}y_{w}z_{w}$ located at point *O* rotates from the intermediate frame with a pitching angle. The pitching axis of the wing is aligned with the leading edge.

According to the quasi-steady (QS) method for aerodynamic analysis of FWRs developed in [14], the instantaneous aerodynamic force $dF_{Aero}\left(r_{w},t\right)$ acting on a 2D wing strip at the span-wise location $r_{w}$ at an instant time *t* is the sum of the translational force $dF_{T}\left(r_{w},t\right)$, rotational force $dF_{R}\left(r_{w},t\right)$, and added mass force $dF_{A}\left(r_{w},t\right)$. Hence, the aerodynamic force of the present wing is expressed in the $y_{w}$ and $z_{w}$ directions as follows:

(1) $$\begin{array}{cc} dF_{Aero,y}\left(r_{w},t\right)= & \;dF_{T,y}\left(r_{w},t\right)+dF_{R,y}\left(r_{w},t\right)+dF_{A,y}\left(r_{w},t\right)\\ \; & =\left\{0.5\rho_{air}{\left|U\right|}^{2}C_{H}cr_{w}+\left(\lambda_{z}u_{z}\omega_{x}-\lambda_{z\omega }{\omega_{x}}^{2}\right)\right\}dr_{w} \end{array}$$

(2) $$\begin{array}{cc} dF_{Aero,z}\left(r_{w},t\right)= & dF_{T,z}\left(r_{w},t\right)+dF_{R,z}\left(r_{w},t\right)+dF_{A,z}\left(r_{w},t\right)\\ & \begin{array}{cc} = & \left\{0.5\rho_{air}{\left|U\right|}^{2}C_{V}cr_{w}+C_{rot}\left|U\right|\omega_{x}{c(r_{w})}^{2}\right\}dr_{w}\\ \; & +\left({-\lambda }_{z}{\dot{u}}_{z}-\lambda_{z\omega }{\dot{\omega }}_{x}\right)dr_{w} \end{array} \end{array}$$

where $\rho_{air}$ is the air density. $\left|U\right|$ is the Euclideam norm of the translational velocity, with $U=r_{w}\omega ={r_{w}\left[\begin{array}{c} \omega_{z},-\omega_{y} \end{array}\right]}^{T}$, which determines the effective angle of attack denoted by $\alpha_{e}=\arctan\left({-\omega }_{y}/\omega_{z}\right)$. $C_{H}$, $C_{V}$, and $C_{rot}$ are empirical coefficients. Specifically, $C_{H}$ and $C_{V}$ are quasi-steady aerodynamic coefficients due to the wing translation motions, while $C_{rot}$ is associated with the pitching motion. $\lambda_{z}$ and $\lambda_{z\omega }$ are the added mass coefficients, given by $\lambda_{z}=\pi /4\rho_{air}{c(r_{w})}^{2}$ and $\lambda_{z\omega }=\pi /4\rho_{air}h{c(r_{w})}^{3}$.

The instantaneous lift *L* and thrust $T_{t}$ of the whole wing are computed by separately integrating $dF_{Aero,z}$ and $dF_{Aero,y}$ for each strip along the wingspan, using the inertial frame $x^{\prime \prime }y^{\prime \prime }z^{\prime \prime }$ as the reference.

The mass of a wing strip is denoted by $dm_{w}$. The inertial force at the $i^{th}$ wing strip from the trailing edge (TE) to the leading edge (LE) in the $y_{w}$ and $z_{w}$ directions are obtained by the following equations:

(3) $$dF_{Iner,y}\left(r_{w},t\right)=\int_{TE}^{LE}\left({\dot{\omega }}_{z}r_{w}+{\omega_{x}}^{2}y_{m}\right)dm_{w}$$

(4) $$dF_{Iner,z}\left(r_{w},t\right)=\int_{TE}^{LE}\left({\dot{\omega }}_{y}r_{w}-{\dot{\omega }}_{x}y_{m}\right)dm_{w}$$

where $y_{m}$ is the chord-wise distance between the $dm_{w}$ and the $z_{w}$ axis. The resulting forces acting on the 2D wing strip are expressed as follows:

(5) $$dF_{y}\left(r_{w},t\right)=dF_{Aero,y}\left(r_{w},t\right)+dF_{Iner,y}\left(r_{w},t\right)$$

(6) $$dF_{z}\left(r_{w},t\right)=dF_{Aero,z}\left(r_{w},t\right)+dF_{Iner,z}\left(r_{w},t\right)$$

The resulting force of the whole wing, $F_{w}\left(t\right)={\left[\begin{array}{c} 0,\;F_{y}\left(t\right),\;F_{z}\left(t\right) \end{array}\right]}^{T}$, is derived by integrating the 2D forces of each strip along the wingspan *R* as follows:

(7) $$F_{y;z}\left(t\right)=\int_{0}^{R}dF_{y;z}\left(r_{w},t\right)$$

### 2.2. Modelling and Dynamic Analysis of the FWR Model

Building on the definition of the aerodynamic model, the dynamic analysis of the FWR model involves determining the kinematics of the flapping and driving mechanisms and formulating the dynamic motion equations. This analysis examines the effects of the driving mechanisms’ size, resonance, and spring stiffness on the power efficiency of the FWR, as discussed in the following sections.

#### 2.2.1. Kinematics of FWR Wing Motions

The flapping motion is assumed to be sinusoidal in most analyses of flapping-wing or FWR MAVs. However, when a DC motor and a crank-slide transmission mechanism drive a loaded flapping mechanism, the motion is no longer purely sinusoidal. Simulating the wing motion of the actual mechanism is crucial for predicting the aerodynamic forces accurately.

**Figure 3:**
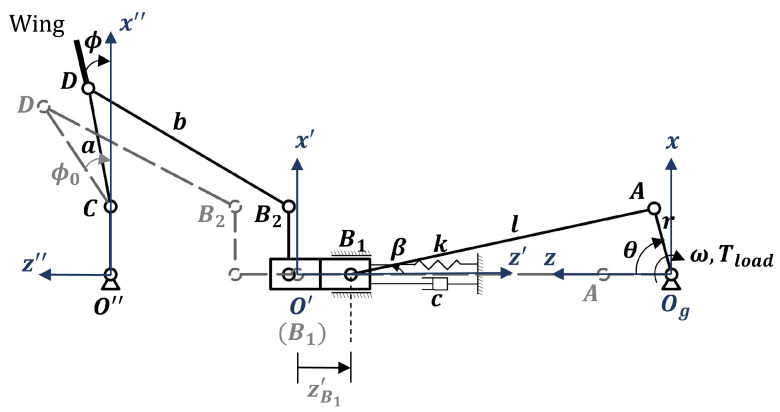
Mechanical system of the FWR model.

The mechanical system of the FWR model, depicted in Figure 1 and further detailed in Figure 3, illustrates the main components of the flapping and driving mechanisms along with their kinematics in detailed coordinate systems. A slider, $B_{1}$, moves linearly along the *z*-axis when driven by a motor through the gears, a crank arm, $O_{g}A$, at an angular speed, ω ($\dot{\theta }$), and a linkage, $AB_{1}$. A spring with stiffness *k* is connected between the $B_{1}$ slider and a fixed point. The bearing of the $B_{2}$ slider moves with the $B_{1}$ slider, linking to the flapping bar CD at point *D* via the linkage bar $B_{2}D$ using pin joints. The length of the $O^{\prime \prime }C$ bar and the distance between $B_{2}$ and the central axis are equal. The grey dotted line and corresponding joint points depict the maximum displacement of the flapping bar motion constrained by the sliders.

The $o_{g}yz$ global coordinate system is established for the kinematics of the geared crank motion, the $o^{\prime }y^{\prime }z^{\prime }$ coordinate system is used to measure slider $B_{1}$’s motion, and the $o^{\prime \prime }y^{\prime \prime }z^{\prime \prime }$ coordinate system is used for the flapping bar. Thus, governed by the instantaneous motion of the geared crank, the flapping motion of the FWR is expressed by the flapping angle ϕ(t) as follows:

(8) $$\begin{array}{cc} \phi \left(t\right) & =z_{B1}^{\prime }\left(t\right)/a-\phi_{0}=r\left[1-\cos\left(\omega t\right)+0.25\lambda \left(1-\cos\left(2\omega t\right)\right)\right]/a-\phi_{0} \end{array}$$

where $z_{B1}^{\prime }$ is the slider motion, λ=r/l is the length ratio of the crank arm *r* to the connecting rod *l*, and $\phi_{0}$ is the initial flapping angle relative to the slider’s limit position, determined by the cosine law. The flapping frequency is f=ω/2π. The flapping amplitude of the FWR model is $65^{\circ }$, with a $+30^{\circ }$/$-35^{\circ }$ flapping angle.

The kinematic description of the passive pitching angle variation of an FWR model has been investigated in previous studies [16,28]. Under the effects of aerodynamic pressure and inertial force, the wing pitching angle α increased rapidly to its maximum at the beginning of the upstroke and remained constant for the rest of the upstroke. Then, during the short period between the end of the upstroke and the downstroke, the wing pitching motion reversed direction and reached its minimum pitching angle. The passive pitch angle of the current FWR with the bifurcated angle limiter varied between $7^{\circ }$ and $40^{\circ }$. Our experimental results indicated that the rotating frequency is approximately equal to the flapping frequency. For rapid estimation of the aerodynamic and inertial forces generated by the model, it was assumed that the rotating velocity remains constant throughout a flapping cycle.

#### 2.2.2. Dynamic Motion Equation of the FWR Model

With gear transmissions, the nonlinear dynamic equation of the DC motor used in our FWR system was taken from previous research in [27,29] as follows:

(9) $$T_{load}+J_{e}\ddot{\theta }+B_{e}\dot{\theta }=K_{u}u$$

where $T_{load}$ is the load torque introduced by the aerodynamic, inertial, and elastic forces; $J_{e}$ is the total effective moment of inertia of the motor and gears; $B_{e}$ is the effective damping coefficient of the motor’s rotating elements; and $K_{u}$ is the linear input gain relative to the gear ratio and the motor torque constant and resistance.

The motor-driven flapping motion is generally excited with a sinusoidal input voltage $u=\bar{U}\sin\left(\Omega t\right)$ [29,30], where $\bar{U}$ is the magnitude and Ω is the motor’s angular velocity. When the crank rotates at a constant angular speed ($\ddot{\Omega }$, $\ddot{\theta }$ = 0), without considering the damping of the motor and gears ($B_{e}$ = 0), the load torque can be directly represented by the motor output torque, $T_{load}=K_{u}u$.

The spring keeps the original length at the halfway point of the slide travel, $z_{B1}^{\prime }=r$. The load torque at the instant time *t* applied to the crank-gear mechanism can be obtained as follows:

(10) $$\begin{array}{cc} T_{\mathrm{load}}\left(t\right)= & \;r\left(\sin\left(\theta +\beta \right)/\cos\beta \right)\left[m_{\mathrm{load}}{\ddot{z}}_{B1}^{\prime }\left(t\right)+k\left(z_{B1}^{\prime }\left(t\right)-r\right)+F_{z^{\prime \prime }}\left(t\right)\right] \end{array}$$

where $m_{load}$ is the load mass; *k* is the spring constant, and when *k* = 0, it indicates the baseline model without springs; and $F_{z^{\prime \prime }}\left(t\right)$ is the resulting force $F_{w}\left(t\right)$ of the FWR wing in the direction of the $z^{\prime \prime }$-axis of the inertial frame. The dimensionless time $\hat{t}$ is defined as $\hat{t}=\omega t$.

#### 2.2.3. Effect of λ on Kinematics of Motion and Aerodynamic Forces

**Figure 4:**
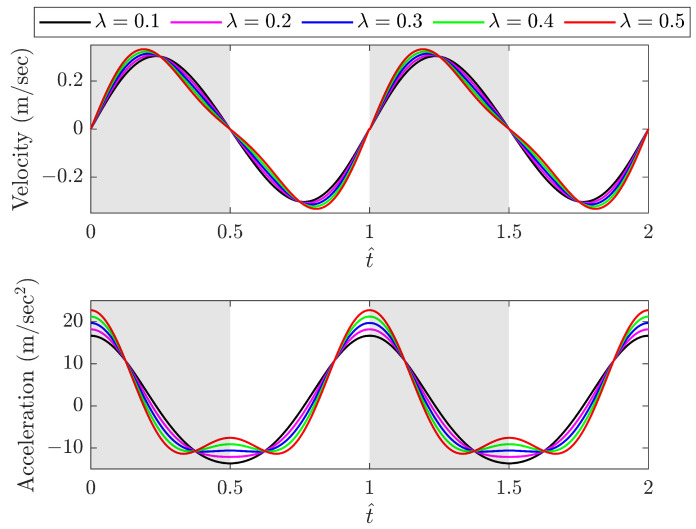
Variation in slide velocity and acceleration with reference to the $o^{\prime }y^{\prime }z^{\prime }$ coordinates for different λ values (grey area: downstroke; white area: upstroke).

The length ratio λ (λ=r/l) significantly impacts the kinematics of the sliding motion. The sliding velocity and acceleration are obtained for different λ values with reference to the $o^{\prime }y^{\prime }z^{\prime }$ coordinates, and the results are shown in Figure 4, where the grey and white areas represent the downstroke and upstroke of the flapping motion, respectively. Given a small angular change Δθ near the starting position at $t_{1}=\Delta \theta /\omega$, the slide velocity is ${\dot{z}}_{B1}^{\prime }\left(t_{1}\right)$=ωr(Δθ+λΔθ), whereas near the end of the wing downstroke at $t_{2}$=(π−Δθ)/ω, the slide velocity is ${\dot{z}}_{B1}^{\prime }\left(t_{2}\right)$=ωr(Δθ−λΔθ). Obviously, ${\dot{z}}_{B1}^{\prime }\left(t_{1}\right)>{\dot{z}}_{B1}^{\prime }\left(t_{2}\right)$ for any non-zero λ, and the slide maximum velocity occurs before one quarter and after three quarters of a period for a whole cycle. A larger λ results in a greater time difference between the positive and negative peaks of velocity in a flapping cycle, leading to increased acceleration at the beginning and end of the cycle. It is clearly shown in Figure 4 that when λ is greater than 0.3, multiple acceleration peaks occur near the midpoint of the cycle as the wing transitions from a downstroke to an upstroke. When λ is small enough to be ignored, the kinematics of the slide and flapping motions can be simplified to simple harmonic motions.

**Figure 5:**
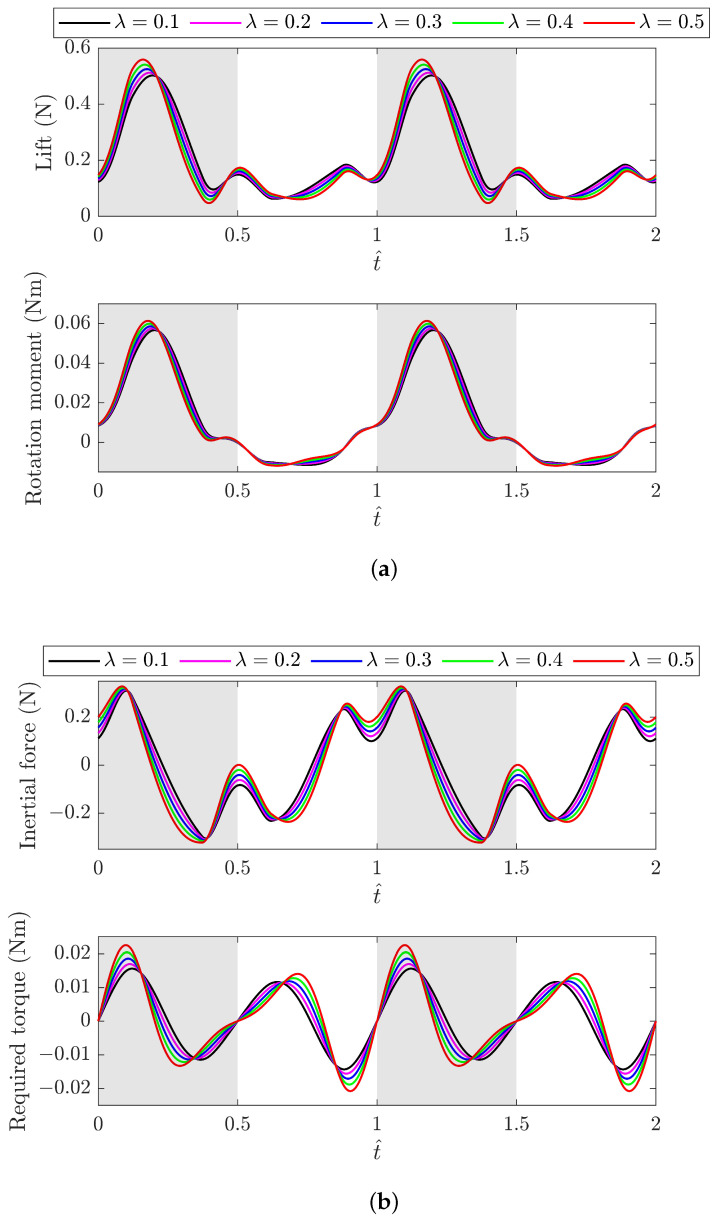
Instantaneous variation in (**a**) lift and rotation moment; (**b**) wing inertial force and required torque for driving the baseline FWR model using different λ values, with reference to the $o^{\prime \prime }y^{\prime \prime }z^{\prime \prime }$ coordinates.

The length ratio further affects the production of aerodynamic forces and the required torque for driving the system instantaneously. The results are obtained and presented in Figure 5 for driving the baseline FWR model using different λ values with reference to the $o^{\prime \prime }y^{\prime \prime }z^{\prime \prime }$ coordinates. Figure 5a shows the results for the lift and rotation moments. A larger λ results in both a higher peak lift and rotation moment during the downstroke. These also occur earlier due to the higher initial acceleration and the earlier velocity peak of the flapping motion. Figure 5b shows the results for the inertial force and required torque. It is obvious that the peak value of the wing inertia force increases with an increasing value of λ, which is consistent with the sliding acceleration variation. Similarly, the variation in the required torque is influenced by both aerodynamic and inertial forces, increasing dramatically with increasing values of λ.

**Figure 6:**
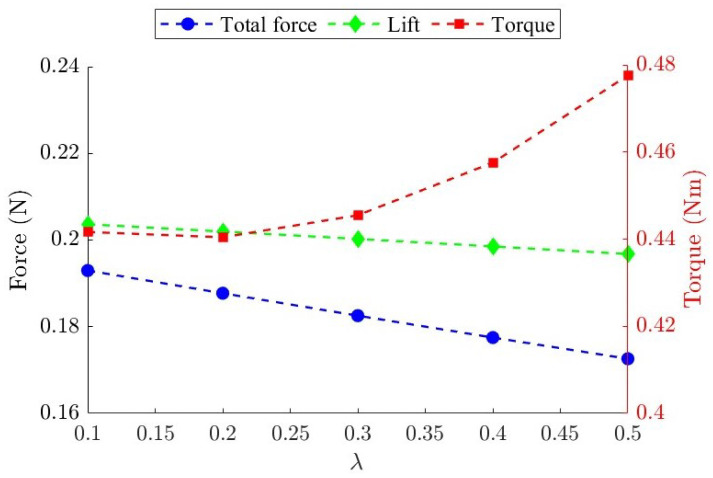
Variation in the average lift, total force, and input torque of the baseline FWR model with different λ values.

Figure 6 presents the average lift, total force, and input torque of the baseline FWR model flapping at 8 Hz with different λ values. It can be seen that the average lift decreases, although a higher λ results in higher peak lift values. The total force, which is the sum of the lift and inertial force, diminishes with a larger λ, because understandably, the inertial force increases and acts in the opposite direction to the declining lift. It is also observed that when λ exceeds 0.3, the increasing inertial force causes the required torque to rise rapidly. Looking at λ=0.5, the FWR requires 7% more driving torque and generates 7.5% less total force compared to when λ is 0.3 while flapping at the same frequency.

**Figure 7:**
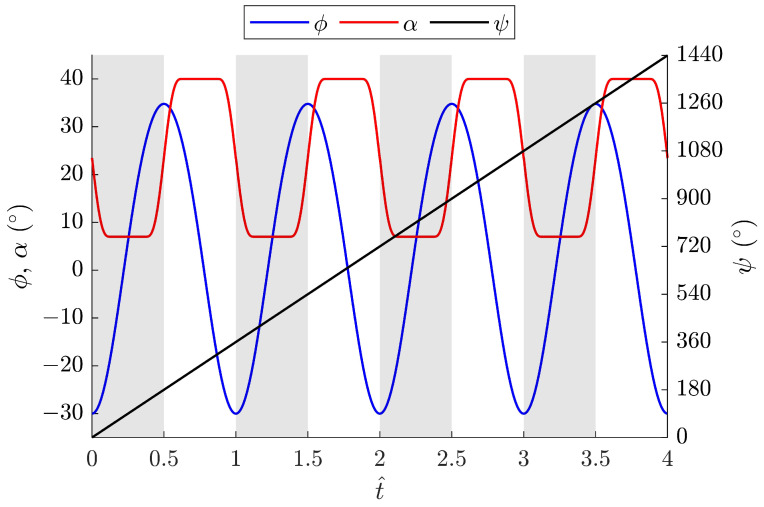
Variation in flapping ϕ, pitching α, and rotation ψ angles of the baseline FWR model with λ=0.22 at a flapping frequency of 8 Hz, with reference to the $o^{\prime \prime }y^{\prime \prime }z^{\prime \prime }$ coordinates.

**Figure 8:**
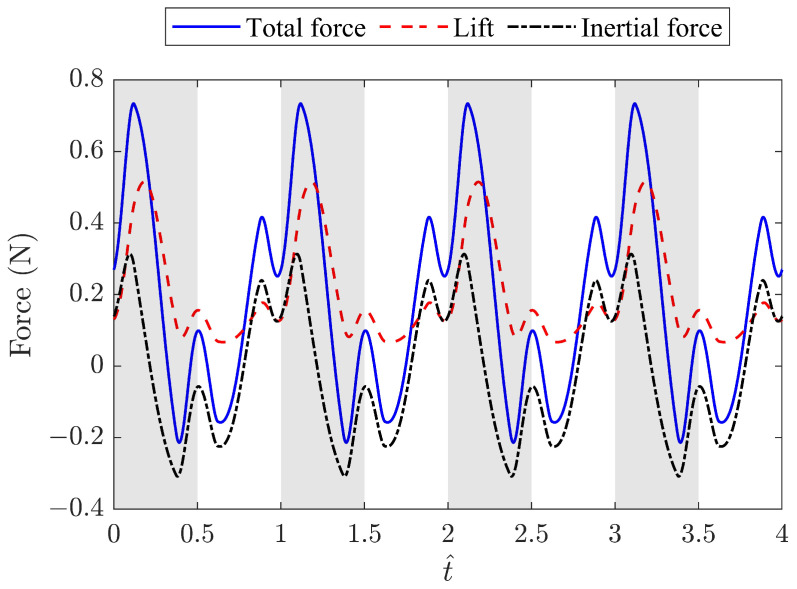
Total force, inertial force, and lift of the baseline FWR model with λ=0.22 at a flapping frequency of 8 Hz, with reference to the $o^{\prime \prime }y^{\prime \prime }z^{\prime \prime }$ coordinates.

A higher λ corresponds to a longer crank arm. To maintain a compact size and minimum power consumption, λ=0.22 was chosen for the FWR test model. The resulting angle variations of the wing flapping, pitching, and rotation motions at 8 Hz are shown in Figure 7. The results for the total force, inertial force, and lift of the baseline FWR model are shown in Figure 8. It is clear that the increase in the passive pitching angle during the upstroke leads to a decrease in forces, especially the lift. More importantly, the maximum and minimum values of the lift and inertial force occur during the transition from downstroke to upstroke when the wing’s pitching angle changes from $7^{\circ }$ to $40^{\circ }$ to produce additional downward thrust.

#### 2.2.4. Effects of Resonance and Spring Stiffness

The wing’s inertial force is proportional to the acceleration ${\ddot{z}}_{B1}^{\prime }\left(t\right)$. The torque $T_{load}$, defined in (10), can be nondimensionalised to ${\hat{T}}_{load}$ as $T_{load}/\left(m_{e}r^{2}{\omega_{n}}^{2}\right)$, where ${m_{e}r}^{2}$ is the mass moment of inertia, and $\omega_{n}$ is the natural frequency.

(11) $$\begin{array}{cc} {\hat{T}}_{load}\left(\hat{t}\right) & =\delta \left(\hat{t}\right)\left\{{(\omega }^{2}/{\omega_{n}}^{2})\left[\cos\left(\hat{t}\right)+\lambda \cos\left(2\hat{t}\right)\right]\right\}\\ & \;\;+\delta \left(\hat{t}\right)\left\{-\cos\left(\hat{t}\right)+0.25\lambda \left(1-\cos\left(2\hat{t}\right)\right)\right\}\\ \; & \;\;+\delta \left(\hat{t}\right)F_{Aero,z}/\left(m_{e}r^{2}{\omega_{n}}^{2}\right) \end{array}$$

The effective mass $m_{e}$ refers to the sum of $m_{load}$ and the wing’s inertial mass. The natural frequency of the system in resonance is predicted by $\omega_{n}=\sqrt{k/m_{e}}$. The expression for $\delta \left(\hat{t}\right)$ can be simplified as follows:

(12) $$\begin{array}{c} \delta \left(\hat{t}\right)=\;\sin\left(\theta +\beta \right)/\cos\beta \approx \sin\left(\hat{t}\right)+0.5\lambda \sin\left(2\hat{t}\right)/(1-0.5\lambda^{2}\sin^{2}\left(\hat{t}\right)) \end{array}$$

The kinematic motion of the FWR test model with a small λ approximates simple harmonic motion. When the system achieves resonance at ${\omega =\omega }_{n}$ without mechanical damping, only the aerodynamic term opposes the torque, as the elastic and inertia forces cancel each other out. In other words, the input torque from the motor is minimised when the FWR operates in resonance, meaning that only the aerodynamic and mechanical damping forces need to be adjusted for.

The power efficiency of the FWR model at a certain flapping frequency can be defined as follows:

(13) $$P_{e}=L/P$$

where *P* is the input power from a motor. In the analytical model, the power efficiency is evaluated by $P_{e}^{*}=L/T_{load}$.

A power-saving value considering the spring effect can be calculated by

(14) $$S_{P}=\left(P_{0}-P_{s}\right)/P_{0}\times 100\left(\%\right)$$

where Ps and Po represent the required power (torque) for the model with and without springs, respectively. The power reduction is expressed by $\Delta P=P_{0}-P_{s}$.

**Figure 9:**
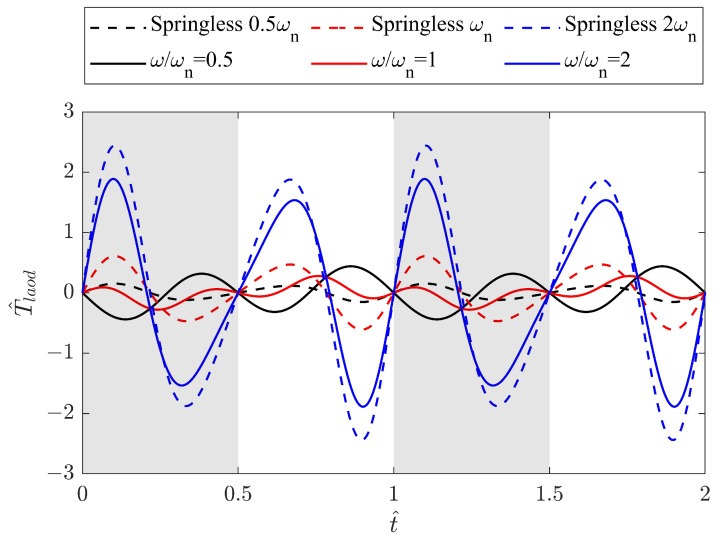
Variation in ${\hat{T}}_{load}$ with dimensionless time $\hat{t}$ in a range of ω for the model with a spring stiffness of k=0.05 N/mm, together with the springless baseline model at $0.5\omega_{n}$, $\omega_{n}$, and $2\omega_{n}$.

**Figure 10:**
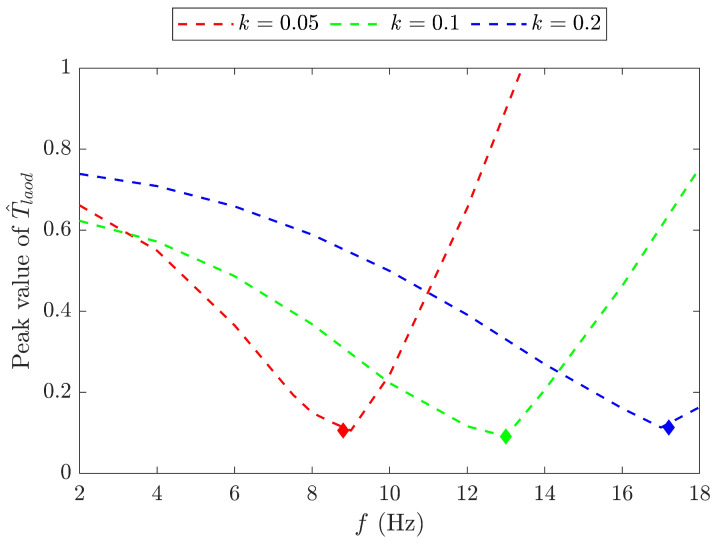
The peak value of ${\hat{T}}_{load}$ versus the flapping frequency *f* with different spring stiffnesses *k*.

**Figure 11:**
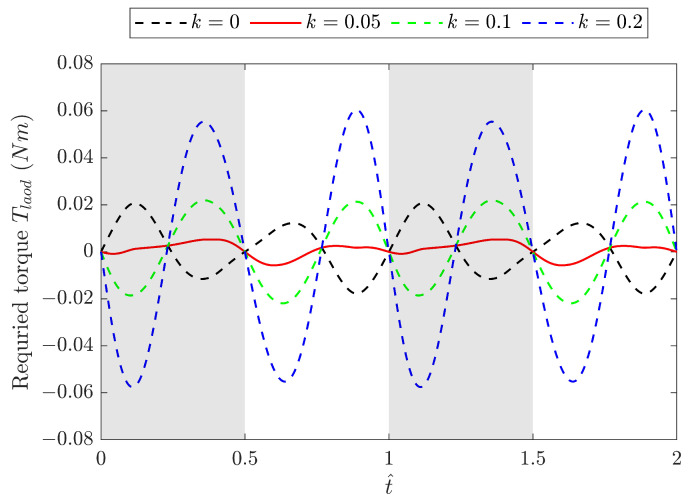
Required torque $T_{load}$ of the FWR model with different spring stiffnesses *k* compared with the baseline model at a flapping frequency of 8 Hz.

- Case 1: constant ω driven by the motor
  Under the condition of a constant ω driven by the motor, the resonance analysis was first conducted on the pure mechanism by excluding the aerodynamic force term $F_{Aero,z}$ in (11). Subsequently, this term was incorporated to evaluate the effect of the aerodynamic force production on the required torque of the FWR mechanical system.

1. Case without aerodynamic forces
  The dimensionless required torque ${\hat{T}}_{load}$ of the pure mechanism with a fixed spring stiffness of k=0.05 N/mm was investigated for various $\omega /\omega_{n}$ values, and the results for three specific cases are shown in Figure 9 against the dimensionless time $\hat{t}$. For comparison, the baseline model without a spring operates at the corresponding frequencies of the spring model in the following cases: $\omega =0.5\omega_{n}$, $\omega_{n}$, and 2$\omega_{n}$. The baseline model results are depicted in the figure using dashed lines. Clearly, at resonance, i.e., $\omega /\omega_{n}$ = 1, ${\hat{T}}_{load}$ is minimised at $\lambda \delta \left(\hat{t}\right)\left(0.25+0.75\cos\left(2\hat{t}\right)\right)$. As a result, the peak value of ${\hat{T}}_{load}$ is reduced by 54% and the power saving is $S_{P}=54\%$ compared to the baseline model at the same frequency $\omega_{n}$. When the frequency exceeds $\omega_{n}$, the required torque increases with the frequency. At $\omega /\omega_{n}=2$, $S_{P}$ reduces to 23%, keeping the torque smaller than the baseline model.
  The ${\hat{T}}_{load}$ is obtained for three different spring stiffnesses, 0.05 N/mm, 0.1 N/mm, and 0.2 N/mm, and the results are shown in Figure 10 against a wide range of frequencies. The natural frequencies of the FWR model are determined as 8.8 Hz, 12.8 Hz, and 17.5 Hz, respectively, from the minimum peak values of ${\hat{T}}_{load}$. This figure also highlights the effect of the spring stiffness on the required torque across the frequency range. It can be seen that models with a lower spring stiffness, such as 0.05 N/mm, require less torque at lower flapping frequencies, whereas a higher stiffness such as 0.2 N/mm requires considerably less torque at higher frequencies.
2. Case with aerodynamic forces
  The required torque was then investigated for three different springs together with the springless baseline model under the condition of aerodynamic force generation. The results are presented in Figure 11 at a flapping frequency of 8 Hz. The aerodynamic force produced by the spring models remains the same as that of the baseline model. The model with a 0.05 N/mm spring has a resonance frequency that is close to 8 Hz, resulting in the smallest required torque compared to other models, while producing the same aerodynamic force. Clearly, the spring stretches before the mid-downstroke, providing an elastic force that aids wing flapping and results in negative torque. After the second half of the downstroke, the spring compresses, leading to an increase in the positive torque. The same pattern reverses for the upstroke.

- Case 2: constant input torque from the motor
  At a given flapping frequency with the same constant ω as in Case 1, the kinematics of the flapping motion remain consistent for the model regardless of the spring’s involvement. Ideally, excessive spring potential energy should be transferred back to the motor for later use [26]. However, it is impractical to save energy directly through a DC motor. Thus, a constant input voltage/torque is assumed for a motor-driven flapping-wing robot [27]. Obviously, a DC motor is unable to maintain a constant rotating speed without a speed controller. In reality, the counter-electromotive force from the DC motor produces a negative torque against the excessive energy and causes an increase in the motor power. Therefore, a constant torque rather than a constant speed ω is the primary consideration when simulating the spring’s effect on both the kinematics of the wing motion and the power requirements.

**Figure 12:**
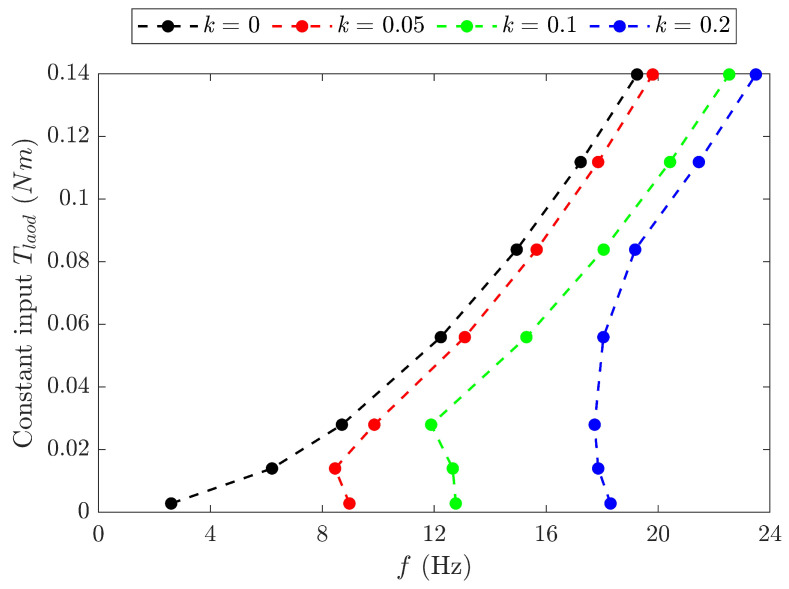
Input torque $T_{load}$ for the FWR model with different spring stiffnesses *k* versus flapping frequencies *f*.

The least squares method was employed to solve the nonlinear dynamic equation of the FWR model represented in (10). The results of the motor input torque are obtained against a range of flapping frequencies, as shown in Figure 12. The minimum input torques for the baseline model and the spring models with stiffnesses of 0.05 N/mm, 0.1 N/mm, and 0.2 N/mm occur at 2.5 Hz, 8.89 Hz, 12.5 Hz, and 17.9 Hz, respectively, aligning with the resonant frequencies of the models. For a fixed input torque, the flapping frequency of the spring models is greater than the baseline model and increases with an increasing spring stiffness. Consequently, the aerodynamic and inertial forces generated by the FWR models with springs are larger than those of the baseline model.

**Figure 13:**
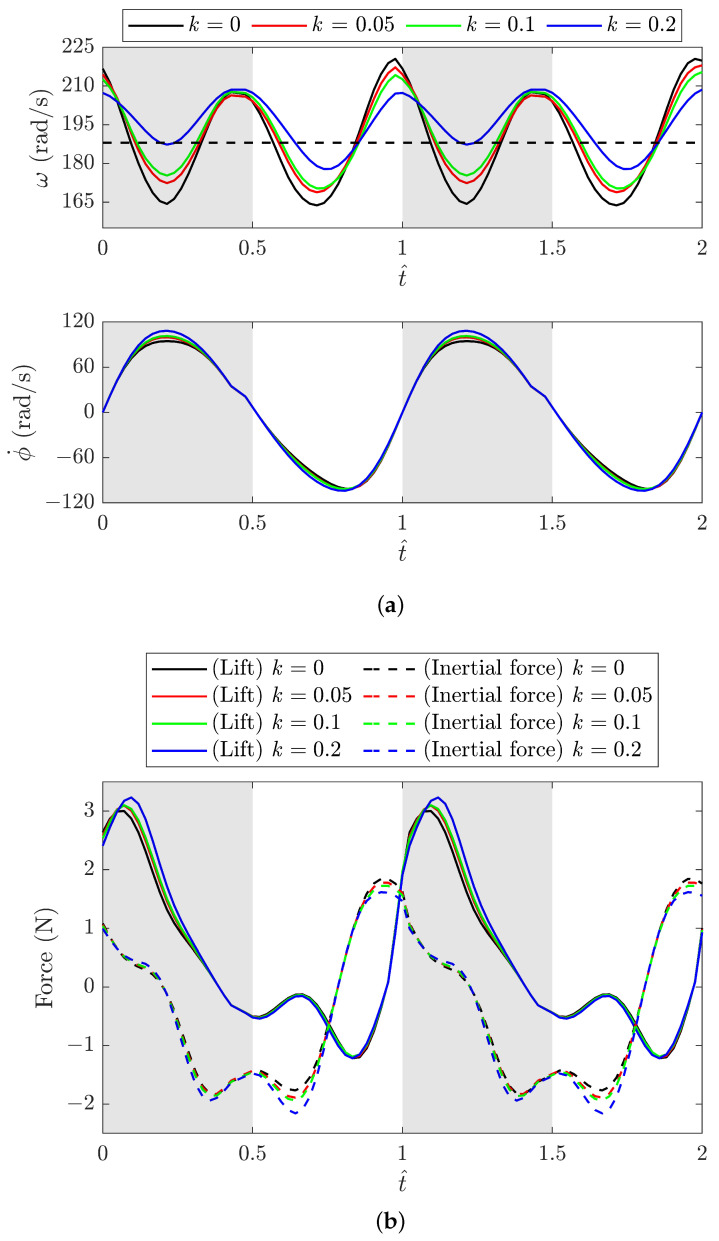
(**a**) Variation in the crank angular speed ω and the wing flapping angular velocity $\dot{\phi }$ with different spring stiffnesses *k* versus $\hat{t}$; (**b**) the lift and inertial force versus $\hat{t}$ (ω with reference to the xyz coordinates; $\dot{\phi }$ and forces with reference to the $x^{\prime \prime }y^{\prime \prime }z^{\prime \prime }$ coordinates).

In this case, each model exhibits distinct kinematics of motion for a constant input torque. Therefore, the kinematic and force results from the spring models and the baseline model are compared in Figure 13 by selecting a time-averaged crank angular velocity ω of approximately 188 rad/s around 30 Hz. Figure 13a indicates that the crank angular speed ω varies periodically, with a smaller amplitude for a larger spring stiffness. A higher ω value near the mid-downstroke noticeably leads to a higher flapping angular velocity $\dot{\phi }$ of the wing. Consequently, the FWR model with the highest spring stiffness of 0.2 N/mm exhibits the largest positive peak $\dot{\phi }$ near the mid-downstroke. This leads to the greatest increase in lift, as shown in Figure 13b. Obviously, the difference in the peak inertial force becomes more pronounced during the upstroke.

**Figure 14:**
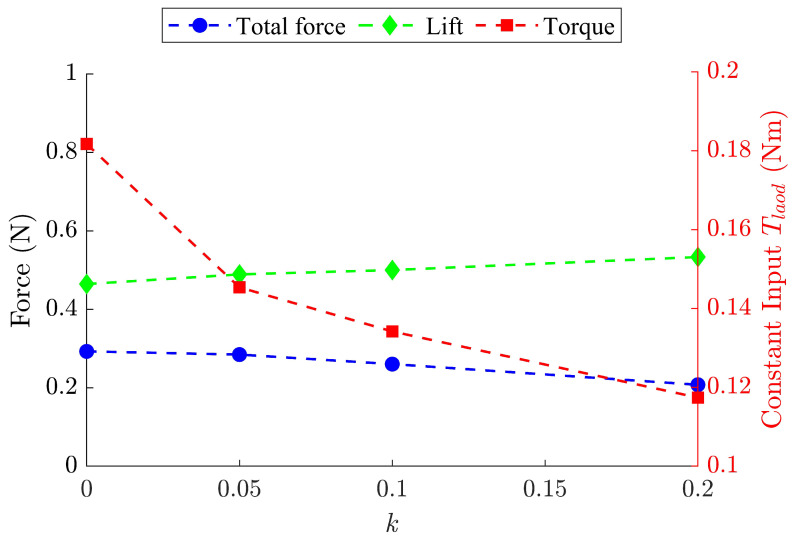
Time-averaged total force, lift, and input torque versus spring stiffness *k*.

Furthermore, the time-averaged results for the lift and total force along with the corresponding constant input torque were obtained, as shown in Figure 14. The aerodynamic lift experiences a slight increase as the spring stiffness increases, while the required torque is significantly reduced. As is obvious, the elastic force generated by the spring plays a major role in actuating the flapping motion. Power savings, $S_{P}$, of 20%, 26%, and 35% are achieved with respect to spring stiffnesses of 0.05 N/mm, 0.1 N/mm, and 0.2 N/mm, compared to the baseline model producing an equivalent lift of approximately 0.5 N. The FWR model with a spring stiffness of 0.2 N/mm achieves a power efficiency $P_{e}^{*}$ of 4.54/m, which is 77% higher than the baseline model of 2.56/m.

In summary, in Section 2, we presented parameter studies of the FWR mechanical system, estimating the system’s resonance frequency and determining the optimal spring stiffness to enhance the design. Subsequent experiments were conducted on the FWR test model with λ=0.22 to validate these analytical results through force and power measurements.

## 3. Experimental Results and Discussion

### 3.1. FWR Model Test Setup

**Figure 15:**
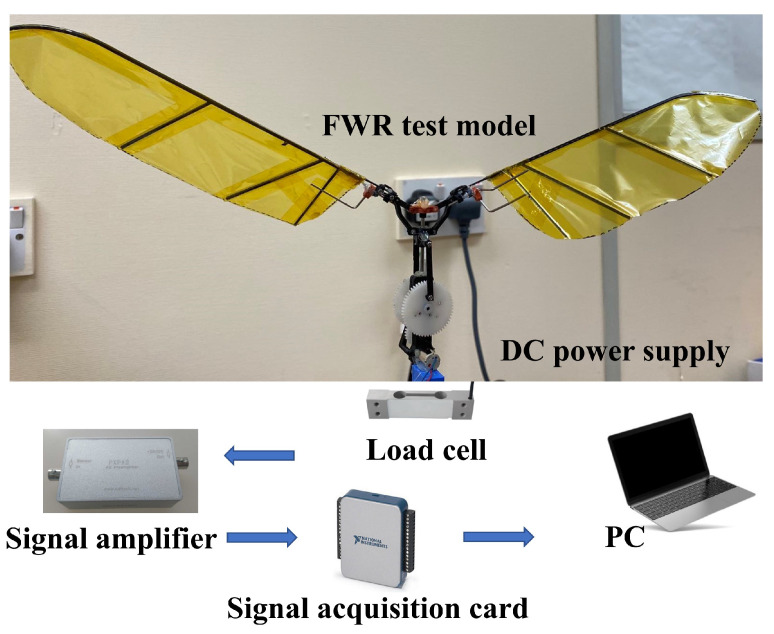
FWR physical model and experimental devices for force measurement.

An experimental platform for the FWR model was constructed using the components depicted in Figure 15 to assess the transverse dynamic force. In order to reflect the analytical results, the FWR model performed the motions as illustrated in Figure 7. The single wing spanned 165 mm, and a maximum chord of 60 mm was chosen. A DC motor drove the FWR model, which was mounted on a high-precision load cell device with a capacity of 200g and inaccuracy of 0.05% F.S. A DC supplier provided the power. The output voltage signal of the load cell was amplified with a gain of 1k and sent to a data acquisition device (NI USB-6009, National Instruments, USA), which was processed using the NI LabVIEW 2018 software to determine the total force generated by the FWR model.

**Figure 16:**
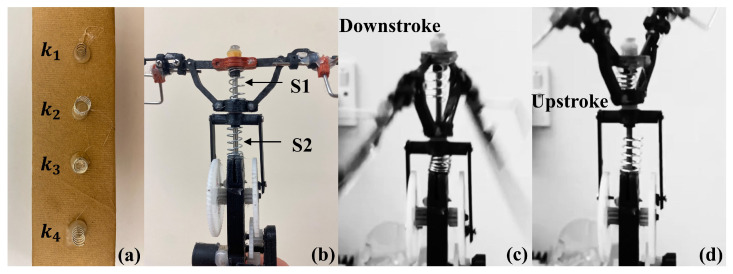
(**a**) Four springs stiffnesses of $k_{1}$ = 0.036, $k_{2}$ = 0.055, $k_{3}$ = 0.11, and $k_{4}$ = 0.22N/mm. FWR model with (**b**) S1 and S2 springs at original lengths, (**c**) lower spring S2 in compression, and (**d**) upper spring S1 in compression.

Based on the analytical study, a range of spring stiffnesses ($k_{1}$ = 0.036, $k_{2}$ = 0.055, $k_{3}$ = 0.11, and $k_{4}$ = 0.22N/mm) were selected for the experiment, as shown in Figure 16. The spring was split into two, S1 above and S2 below the slider. When the slider was in the middle position, with $\theta =90^{\circ }$, both springs were in their neutral position with their original lengths. When the slider moved to the second half of the downstroke (including the wing downstroke), the lower spring S2 compressed. The spring returned to neutral after the first half of the upstroke and then compressed in the second half. The spring arrangement represents the analytical model. In addition, two different mechanisms were tested to reflect the analytical cases: one is a pure mechanism without aerodynamic force generation, while the other includes it.

### 3.2. Test of the Pure FWR Mechanism

**Figure 17:**
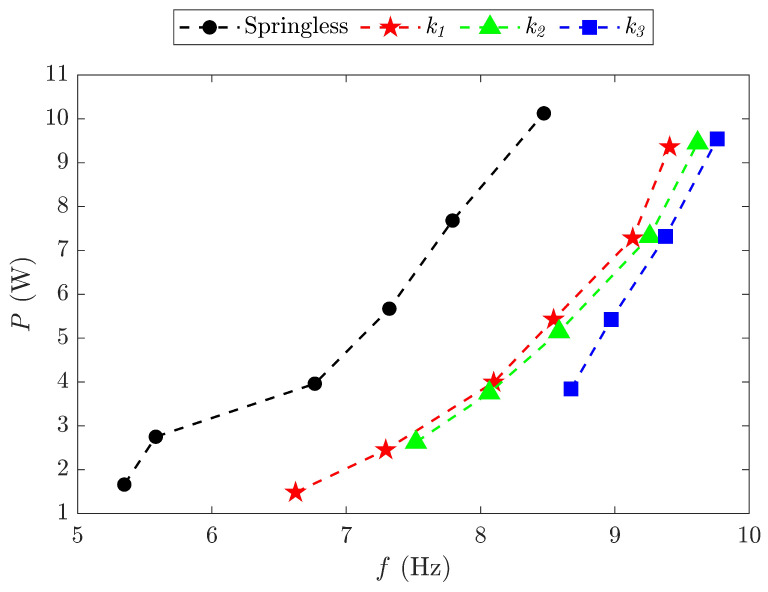
Input power *P* for the FWR mechanical system versus the flapping frequency *f* for different spring stiffnesses *k*.

The wing skin membrane was removed from the FWR mechanical system to eliminate the aerodynamic force effect. The experiment focused on a range of input powers from 1.5 W to 10 W with flapping frequencies from 5 Hz to 10 Hz. The FWR mechanical system was ineffective at the tested low flapping frequency range due to the higher power requirements for $k_{4}$. Therefore, the experiment focused on samples using the lower spring stiffness values of $k_{1}$ to $k_{3}$. The input power in relation to the frequency was obtained as shown in Figure 17. Clearly, the system with springs required less input power than the springless baseline model at any flapping frequency. The initial input power needed to drive the model increased with the spring stiffness, as well as with the flapping frequency.

**Figure 18:**
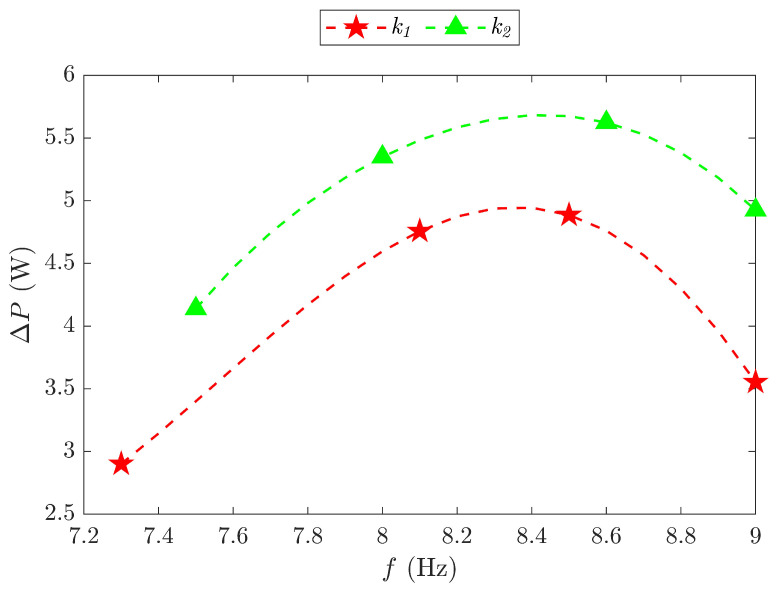
Power reduction ΔP versus the flapping frequency *f* for the $k_{1}$ and $k_{2}$ models.

The power reduction ΔP for the $k_{1}$ and $k_{2}$ models was further investigated within the investigated frequency range, as shown in Figure 18. The peak power reductions were observed at 8.35 Hz for the $k_{1}$ model and 8.45 Hz for the $k_{2}$ model, corresponding to their resonance frequencies. In contrast, the theoretical resonance frequencies calculated by using the analytical method were 7.85 Hz for the $k_{1}$ model and 9.26 Hz for the $k_{2}$ model. The differences between the experimental and theoretical results were 6.37% for the $k_{1}$ model and 8.75% for the $k_{2}$ model, indicating that the analytical method achieved good accuracy. This can also be seen from the experimental results that the maximum reduced input power values for the two models were 4.94 W and 5.68 W, with power savings $S_{P}$ of 49.4% and 53%, respectively.

### 3.3. Test of the FWR Including Aerodynamic Forces

**Figure 19:**
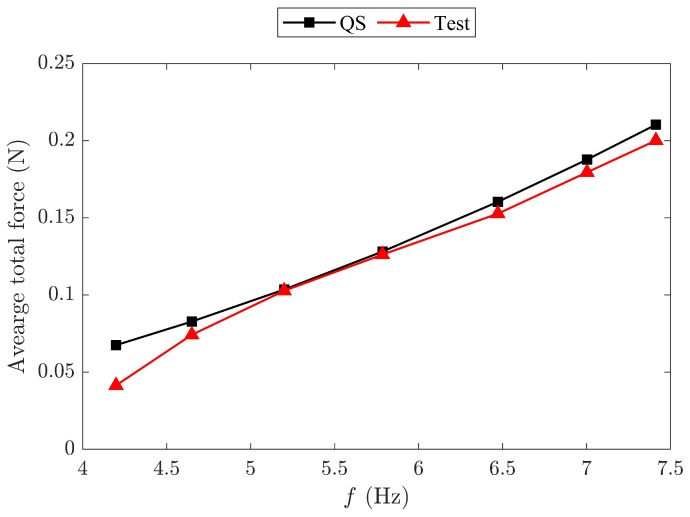
Comparison of the analytical aerodynamic lift results from the QS method with the measured average total force for the springless baseline model.

For this test, the wings were covered with a membrane skin to generate aerodynamic forces. The measured instantaneous total force of the FWR model included both aerodynamic and inertial forces. The average total force measured for the springless baseline model over a flapping cycle was compared to the average aerodynamic lift predicted by the QS method across different flapping frequencies, as shown in Figure 19. Since the average inertial force over a flapping cycle equals zero, the measured total force closely matched the analytical average aerodynamic lift result.

**Figure 20:**
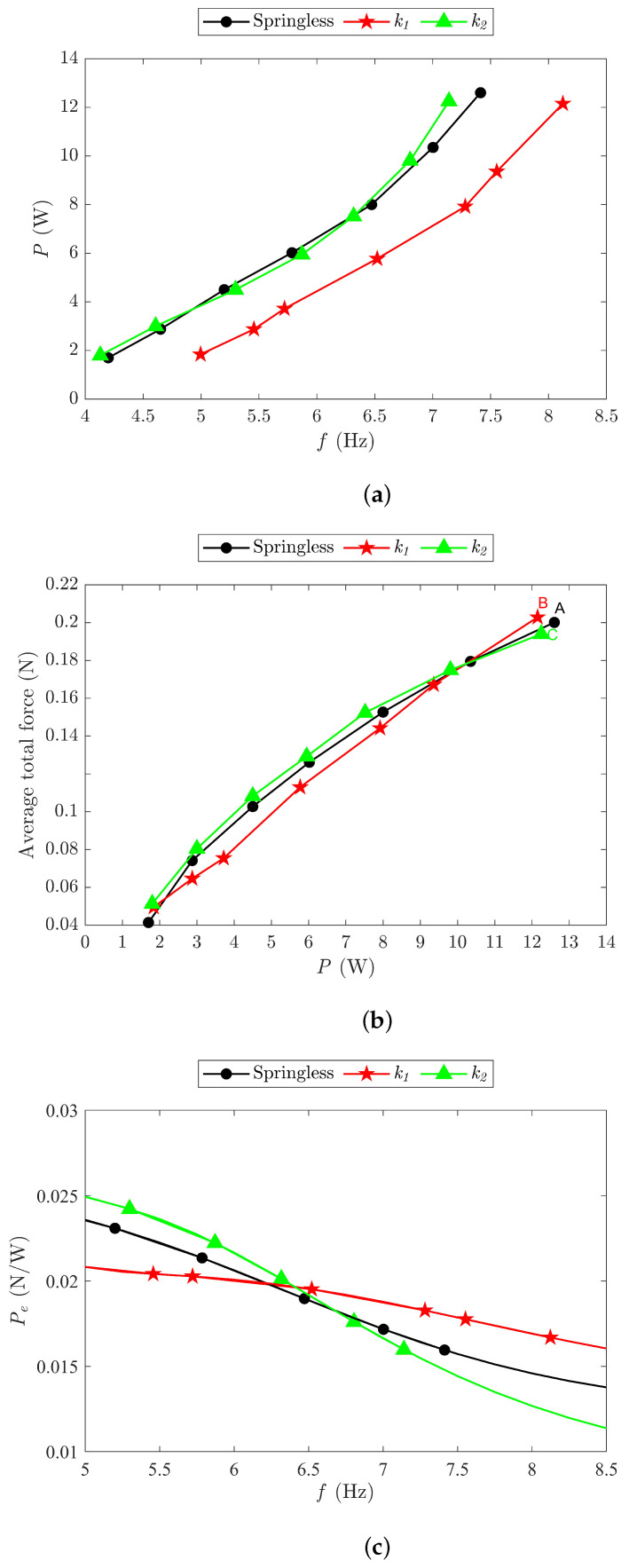
(**a**) Required input power *P* for FWR test model generating aerodynamic force with $k_{1}$ and $k_{2}$ springs versus flapping frequency *f*. (**b**) Measured average total force versus input power *P*. (**c**) Power efficiency $P_{e}$ versus flapping frequency *f*.

The power required to drive the FWR model with aerodynamic force production, using the $k_{1}$ and $k_{2}$ springs, was evaluated across a flapping frequency range of 4 to 8.5 Hz, and the results are presented in Figure 20a. Compared to the results obtained from the pure springless model shown in Figure 17, it can be seen that the power required for producing aerodynamic forces is approximately double. The input power for the springless baseline and $k_{2}$ models increased almost linearly from 4 to 6.5 Hz, followed by a sharper rise beyond that point. The $k_{1}$ model followed a similar trend, exhibiting a steeper increase until 7.3 Hz. The required power for the $k_{1}$ model was about half of that of the baseline and $k_{2}$ models. The power demands for the $k_{3}$ and $k_{4}$ models exceeded the motor capabilities, so no data were obtained.

The average total force was determined for the models of springless, $k_{1}$, and $k_{2}$ against the input power, and the results are shown in Figure 20b. Clearly, the $k_{1}$ model exhibited a slightly lower average total force than the baseline and $k_{2}$ models in the 2 to 10.35 W input power range. The average total forces of the three models at points A, B, and C in the figure are 20.4 g, 20.7 g, and 19.8 g, respectively, with an input power of about 12 W and a DC supply voltage of 5 V. Hence, the FWR test model weighing 19.74 g can achieve vertical take-off.

The power efficiency, $P_{e}$, for the FWR test model was determined using (13), i.e., the average total force-to-input-power ratio, and the results are shown in Figure 20c. It can be observed that the power efficiencies of three models decreased with increasing flapping frequencies from 5 Hz to 9 Hz, with the $k_{1}$ model exhibiting the smallest reduction rate. Interestingly, the $k_{2}$ model demonstrated greater efficiency when the flapping frequency was below 6.5 Hz, which is attributable to the increased aerodynamic force resulting from larger acceleration due to a greater elastic force, despite higher power requirements for spring deformation. However, for any frequency above 6.5 Hz, the power that was required to deform the spring significantly increased, making the $k_{2}$ model less efficient than the $k_{1}$ model. Clearly at 8.1 Hz, the power efficiency of the $k_{1}$ model was 0.017 N/W, which was 16% higher than the baseline model and 33% higher than the $k_{2}$ model.

## 4. Take-Off Flight of the FWR-MAV with Onboard Power

**Figure 21:**
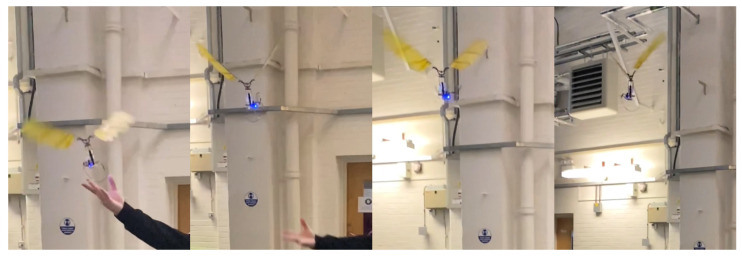
Untethered vertical take-off flight of the FWR-MAV with an onboard power unit.

To demonstrate the feasibility of the FWR model’s flight, the FWR test model shown in Figure 15 was equipped with an onboard power unit including a battery and a control receiver for flight testing. The model components and DC motor details are provided in Figure 1 and Table 1. The FWR-MAV had a total take-off weight of 27.8 g, including a 6.05 g lithium battery (3.7 V, 150 mAh, 25C max discharge current) and a 2 g flight control device. Considering the battery’s maximum operating current of 3.75A, the FWR-MAV requires 13.9 W for take-off. The required input power and flapping frequency are slightly above the experimental range of 13 W in Figure 20. The vertical take-off of the FWR-MAV is shown in Figure 21.

**Figure 22:**
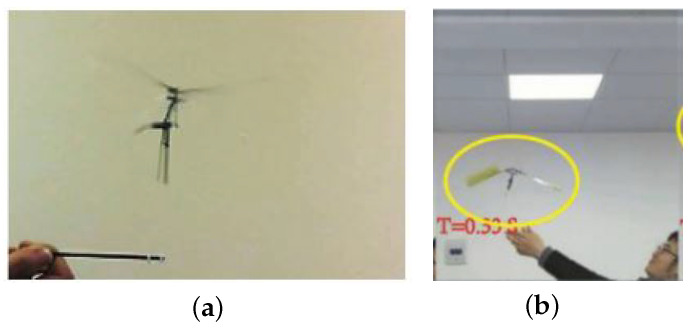
Tethered vertical flight of (**a**) the 2.6 g FWR prototype model [13] and (**b**) the 22.7 g FWR model [16].

Compared to the initial 2.6 g FWR prototype model that achieved vertical take-off in 2017 [13], shown in Figure 22a, the current flight test model has nearly doubled its wing area and generated ten times greater lift. Regarding power efficiency, the current FWR-MAV model achieved 0.02 N/W. For a previous tethered flyable model [16] with a similar weight of 22.7 g without an onboard battery, shown in Figure 22b, the coreless motor had a continuous power of 15 W and a rated voltage of 7.4 V. Since 9V from a DC supply was used for take-off with wires, the input power exceeded the motor-rated power. Based on the rated current, at least 18.24 W was needed for the 22.7 g model to take off, resulting in a power efficiency of 0.012 N/W. Compared to this, the power efficiency of the current FWR-MAV model has improved by 66.7%.

## 5. Conclusions

This study demonstrates the significant enhancement of FWR power efficiency that can be achieved by using an elastic flapping mechanism with springs, employing both analytical and experimental approaches to test a potentially flyable FWR model. The resonant frequency is determined based on the mass distribution and spring stiffness of the mechanical system. The analytical model emphasises that the input power can be reduced by 50% at resonance in comparison to the baseline system without a spring. Furthermore, incorporating an optimally stiff spring can lead to a 20% to 35% reduction in power consumption and an increase in power efficiency of up to 77% compared to the baseline model at a high flapping frequency of around 30 Hz.

The experimental test demonstrates that additional power is required for lift generation compared to the purely mechanical system without skin, yielding the following key findings:

· Incorporating a spring stiffness of 0.036 N/mm in the FWR model results in a maximum input power reduction of 14% and a power efficiency increase of 16% at a flapping frequency of 8.1 Hz (approaching resonance) compared to the baseline model.
· The model achieved 20% less power consumption and a 66.7% increase in power efficiency compared to a previous FWR model of the same weight and size.
· Free flight was successfully achieved with an onboard power unit and remote-control device, demonstrating significant potential for increasing the flight endurance and payload capability in future applications of FWR-MAVs.

## Figures and Tables

**Table 1:** Data on the main components of the FWR model.

Component	Material	Weight (g)	Quantity
Motor	-	4.87	1
Model frame	Polyamide (nylon plastic)	2.76	1
Gear set (4 gears)	Plastics	3.17	1
Rocker	Carbon fibre	0.15	2
Connecting rod and bearing	Carbon fibre, aluminium	0.45	2
Slider and bearing	Aluminium	1.9	1
Bifurcated unit	Aluminium	0.69	2
Vertical shaft	Aluminium	0.9	1
Flapping bar	Carbon fibre	0.3	2
V-linkage	Carbon fibre	0.1	2
Wing rotary bearing	Aluminium	0.3	1
Wing skin	Polyimide	1.33	2
Total	-	19.74	18

## Data Availability

The original contributions presented in this study are included in the article. Further inquiries can be directed to the corresponding author.

## Abbreviations

The following abbreviations are used in this manuscript:

MAV	Micro Aerial Vehicle
FWR	Flapping-Wing Rotor
$x^{\prime \prime }y^{\prime \prime }z^{\prime \prime }$	Inertial frame
$x_{c}^{\prime \prime }y_{c}^{\prime \prime }z_{c}^{\prime \prime }$	Co-rotating frame
$x_{w}y_{w}z_{w}$	Wing-fixed frame
$x_{1}^{\prime \prime }y_{1}^{\prime \prime }z_{1}^{\prime \prime }$, $x_{2}^{\prime \prime }y_{2}^{\prime \prime }z_{2}^{\prime \prime }$	Intermediate frames
*R*	Ratio of wing span
$\bar{c}$	Mean chord
*h*	Distance between pitching axis and leading edge
$c(r_{w})$	Wing strip at span-wise location
ψ, ϕ, α	Angle of rotation, flapping, and pitching
*t*, $\hat{t}$	Instant time, dimensionless time
U, ω	Translational velocity, angular velocity
$\rho_{air}$	Air density
$\alpha_{e}$	Effective angle of attack
$C_{H}$, $C_{V}$	Quasi-steady aerodynamic coefficients of wing translation
$C_{rot}$	Quasi-steady aerodynamic coefficient of wing pitching
$\lambda_{z}$, $\lambda_{z\omega }$	Added mass coefficients
*L*, $T_{t}$	Instantaneous lift and thrust
LE, TE	Leading edge and trailing edge
$dm_{w}$	Mass of a wing strip
$F_{Aero}$, $F_{T}$, $F_{R}$	Instantaneous aerodynamic force, translational force, and rotational force
$F_{A}$	Instantaneous added mass force
$F_{Iner}$	Inertial force
$F_{w}$	Resulting forces of wing strip
ω	Flapping angular speed
θ, $\dot{\theta }$	Rotation angle and angular speed of crank arm

*k*	Spring stiffness
$z_{B1}^{\prime }$	Slide travel
*r*	Crank arm length
*l*	Connecting rod length
λ	Ratio of crank arm length to connecting rod length
$\phi_{0}$	Initial flapping angle
$T_{load}$	Load torque caused by aerodynamic, inertial, and elastic forces
${\hat{T}}_{load}$	Dimensionless torque
$J_{e}$	Total effective moment of inertia of motor and gears
$B_{e}$	Effective damping coefficient of motor rotating elements
$K_{u}$	Linear input gain relative to gear ratio and motor torque constant and resistance
*u*, $\bar{U}$	Sinusoidal input voltage and magnitude
Ω	Motor angular velocity
$m_{load}$, $m_{e}$	Mass of load, effective mass
$\omega_{n}$	Natural frequency
*P*, $P_{e}$, $S_{p}$	Input power from a motor, power efficiency, power-saving value
$P_{s}$, $P_{o}$	Required power (torque) for model with and without spring, respectively
ΔP	Power reduction
